# Actin Crosslinking Family Protein 7 Deficiency Does Not Impair Hearing in Young Mice

**DOI:** 10.3389/fcell.2021.709442

**Published:** 2021-11-30

**Authors:** Benjamin L. Gilbert, Shaoyuan Zhu, Ahlam Salameh, Shenyu Sun, Kumar N. Alagramam, Brian M. McDermott

**Affiliations:** ^1^Department of Otolaryngology–Head and Neck Surgery, Case Western Reserve University School of Medicine, Cleveland, OH, United States; ^2^Department of Biology, Case Western Reserve University, Cleveland, OH, United States; ^3^Department of Genetics and Genome Sciences, Case Western Reserve University School of Medicine, Cleveland, OH, United States; ^4^Department of Neurosciences, Case Western Reserve University School of Medicine, Cleveland, OH, United States

**Keywords:** hair cell, hearing, MACF1, microtubule-actin crosslinking factor 1, ACF7, ABR (auditory brainstem response), cuticular plate

## Abstract

To enable hearing, the sensory hair cell contains specialized subcellular structures at its apical region, including the actin-rich cuticular plate and circumferential band. ACF7 (actin crosslinking family protein 7), encoded by the gene *Macf1* (microtubule and actin crosslinking factor 1), is a large cytoskeletal crosslinking protein that interacts with microtubules and filamentous actin to shape cells. ACF7 localizes to the cuticular plate and the circumferential band in the hair cells of vertebrates. The compelling expression pattern of ACF7 in hair cells, combined with conserved roles of this protein in the cytoskeleton of various cell types in invertebrates and vertebrates, led to the hypothesis that ACF7 performs a key function in the subcellular architecture of hair cells. To test the hypothesis, we conditionally target *Macf1* in the inner ears of mice. Surprisingly, our data show that in young, but mature, conditional knockout mice cochlear hair cell survival, planar cell polarity, organization of the hair cells within the organ of Corti, and capacity to hear are not significantly impacted. Overall, these results fail to support the hypothesis that ACF7 is an essential hair cell protein in young mice, and the purpose of ACF7 expression in the hair cell remains to be understood.

## Introduction

In mammals, the mechanosensitive hair cells in the cochlea of the inner ear are responsible for hearing (Hudspeth, 2005). These cells convert mechanical stimuli associated with pressure changes in the air into electrical responses that are forwarded to the brain. In the cochlea, hair cells are arranged into three rows of outer hair cells (OHCs) and one row of inner hair cells (IHCs) in a particular polarity axis, establishing a specific planar cell polarity (PCP) (May-Simera and Kelley, 2012; Ezan and Montcouquiol, 2013). Disruption of hair cell PCP, ototoxic or noise-induced damage to hair cells, or a decrease in their numbers cause sensorineural hearing loss (Petit and Richardson, 2009; Walsh et al., 2010; Lu and Sipe, 2016).

Hair cells each have a precise array of actin-based stereocilia that insert into a dense actin-based meshwork known as the cuticular plate (Pollock and McDermott, 2015). Proteins that shape the stereocilia are required for hearing and balance (Petit and Richardson, 2009; Richardson et al., 2011). Furthermore, proteins that regulate actin and microtubule filaments may be essential for establishing the cuticular plate and PCP (Goodrich and Strutt, 2011; Copley et al., 2013). Yet, little is known about the roles the proteins that shape the cuticular plate play in hearing and balance. Moreover, the function of the cuticular plate itself with regard to hair bundle development, maintenance, and function is unclear. One potential function is that the cuticular plate provides rigidity to the apical surface of the hair cell to anchor the stereocilia in place (Pollock and McDermott, 2015; Pacentine et al., 2020).

Actin crosslinking family protein 7 (ACF7), encoded by mouse microtubule actin cross-linking factor 1 (*Macf1*), is a member of the spectraplakin family of proteins and contains an F-actin-binding domain near the N-terminus and a microtubule-binding domain near the C-terminus (Suozzi et al., 2012; Goryunov and Liem, 2016; Hu et al., 2016; Zhang et al., 2017). ACF7 is widely expressed across the tissues of metazoans, displaying significant evolutionary conservation (Voelzmann et al., 2017). Previously, we demonstrated in both zebrafish and mouse hair cells that ACF7 reliably localizes to the cuticular plate, the circumferential band, and additionally coats the lumen of the fonticulus, a region that holds the basal body, an organelle subjacent to the kinocilium (Antonellis et al., 2014). The subcellular localization of ACF7 and its ability to bind both actin and microtubule filaments suggest that ACF7 could be involved in integrating cytoskeletal elements to apical structures, a union that may be essential for the cell’s integrity and function. ACF7 additionally may be necessary in the formation and maintenance of the cuticular plate (Kodama et al., 2003; Brown, 2008). Recent studies on conditionally targeted mice using a *Foxg1-Cre* driver and floxed *Macf1* showed that ACF7 is required for normal kinocilium length in the cochlea (May-Simera et al., 2016). However, since these mice die at E18.5, it is unknown if ACF7 is required in the mature mouse for hair cell structure or hearing.

Herein, we investigate the role of ACF7 in hearing and the hair cell’s structure and function by conditionally targeting ACF7 in mouse hair cells. Our results suggest that a deficiency in ACF7 does not significantly impact hair cell structure or function.

## Materials and Methods

### Mouse Husbandry

Protocols for housing and handling of mice were approved by Case Western Reserve University’s Institutional Animal Care and Use Committee. CD1 *Pax2-Cre* [Tg(Pax2-cre)1Akg] mice (Ohyama and Groves, 2004) were obtained from the Mutant Mouse Resource & Research Center (stock #10569). We used *Macf1*^*fl/–*^ (Wu et al., 2008), *Macf1^*fl/+*^, Macf1*^*fl/fl*^, and *Macf1^+/–^; Pax2-Cre* mouse lines. Each strain was outcrossed with FVB/NJ mice for at least seven generations. Specifically, C57BL/6 *Macf1*^*fl/fl*^ mice were outcrossed to FVB/NJ mice (Wu et al., 2008). In addition, to improve the likelihood of Cre-mediated recombination in the cochlea, we generated a germline transmissible knockout allele by crossing *Macf1*^*fl/+*^ with BALB/c *CMV-Cre* mice (Tg(CMV-Cre)1Cgn/J obtained from the Jackson Laboratory stock #003465), with resulting *Macf1^+/−^; CMV-Cre* offspring outcrossed to FVB/NJ mice (Schwenk et al., 1995) for more than seven generations before use in generating experimental data. Note, early publications of *Macf*1^*fl*^ describe *loxP* sites flanking exons 6 and 7 (Wu et al., 2008); however, after modern genomic analyses, the *loxP* sites were shown to flank exons 11 and 13. Ai3-YFP [*Gt(ROSA)26Sor^tm 3(CAG–EYFP)Hze^*] (Madisen et al., 2010) reporter mice were crossed to *Pax2-Cre* mice. One-month old or P5 mice of either sex were used for experiments.

### Mice Genotyping

Mouse genotypes were determined by a standard PCR amplification procedure (Truett et al., 2000). Floxed and wildtype *Macf1* alleles were genotyped using forward primer (F1′: 5′-AAAGAAACGGAAATACTGGCC-3′; exon 10) and reverse primer (R1′: 5′-GCAGCTTAATTCTGCCAAATTC-3′; exon 11). T_A (annealing)_ = 56°C; T_E (extension)_ = 1 min. The *Macf1* knock out (KO) allele was separately genotyped using the forward primer F1′ with reverse primer targeting exon 14 (R2′: 5′-AAAGAAACGGAAATACTGGCC-3′; T_A_ = 50°C; T_E_ = 2.5 min) (Fassett et al., 2013). The *Pax2-Cre* allele was genotyped with the primer pair F′ (5′-GCCTGCATTACCGGTCGATGCAACGA-3′) and R′ (5′-GTGGCAGATGGCGCGGCAACACCATT-3′). T_A_ = 67°C; T_E_ = 1 min (Truett et al., 2000). PCR was conducted in a thermocycler (Applied Biosystems^TM^ SimpliAmp^TM^ Thermal Cycler and on a Bio-Rad PTC-200 DNA Engine^®^ Cycler).

### Cochlear Labeling and Imaging

One-month old mice cochleae were dissected and fixed in 4% paraformaldehyde in 1× phosphate-buffered saline (PBS) overnight at 4°C. Apical, middle, and basal turns of cochleae were defined according to Supplementary Figure 1 (Gilels et al., 2017). The tissue was rinsed three times in 1× PBS for 10 min each and decalcified with 1 mM ethylenediaminetetraacetic acid (EDTA) at 4°C for 2–3 days. Cochleae were blocked and permeabilized simultaneously with 0.05% Triton-X100 (Sigma) and 10% goat serum solution in 1× PBS for 2 h at room temperature. To visualize cells, the tissue was washed and labeled with anti-myosin7a (Proteus Biosciences, Inc.) primary antibody at a 1:500 dilution with blocking solution at 4°C overnight. After three, 5-min washes in blocking solution, tissue was labeled with secondary antibodies at a 1:1,000 dilution for Alexa Fluor 488 IgG (Invitrogen) and a 1:70 dilution for Alexa Fluor 633 phalloidin (Invitrogen) at 4°C overnight, washed, mounted (ProLong Diamond Antifade Mountant), and imaged on a TCS SP8 confocal microscope (Leica). Ai3-YFP/*Pax2-Cre* mice were imaged at an excitation wavelength of 512 nm for YFP.

Whole-mount immunolabeling of cochleae was performed with anti-ACF7 (Karakesisoglou et al., 2000) and mouse anti-acetylated tubulin (T6793; Sigma) as described by Pataky et al. (2004), with modifications being a 1 h fixation, 2 h permeabilization, and 2 h blocking (1% bovine serum albumin) (Pataky et al., 2004). Anti-ACF7 rabbit polyclonal antiserum against amino acids 1,622–1,817 of a partial sequence of the primary ACF7 isoform was used (Bernier et al., 1996; Karakesisoglou et al., 2000). Anti-ACF7 and anti-acetylated tubulin were used at a 1:100 dilution overnight at 4°C. Treatment with goat anti-rabbit and anti-mouse Alexa Fluor 488 IgG (1:200) and Alexa Fluor 633 phalloidin (1:50) occurred for 2 h at room temperature. Mounting and imaging were conducted as above.

### Cell Enumeration and Organelle Length Measurement

For hair cell enumeration, outer and inner hair cells were manually counted using LAS X software (Leica). A hair cell was considered present when the hair bundle and cuticular plate were observed and myosin7a signal detected (Raphael and Altschuler, 1991; Anttonen et al., 2014; Monzack et al., 2015). We used the hair cells from the entire cochlea for calculations. To calculate the percent of present hair cells we used: % hair cells present = present hair cells/(missing + present hair cells) × 100. For organelle length measurements, we measured kinocilia of all useable cells from the apical turn in a confocal series similar to methods described by May-Simera et al. (2016). Kinocilium length measurements of hair cells labeled with anti-acetylated tubulin in P5 mice were obtained with LAS X software (Leica).

### Auditory-Evoked Brainstem Response (ABR)

Mice of one month of age were anesthetized with a ketamine hydrochloride and xylazine hydrochloride solution (100 and 20 mg/kg, respectively) via intraperitoneal injection, and kept on a homeothermic heating pad at 37°C for the duration of sedation. Platinum subdermal needle electrodes were placed below the pinna of the left and right ears to record the ABRs, and on the back of the mice for electrical grounding (Akil et al., 2016; Geng et al., 2017). Electrical responses of the cochlear ganglion neurons and the nuclei were recorded at different levels of pure tone frequencies played for 100 ms at 8, 16, and 32 kHz (Intelligent Hearing System SmartEP 4.20 system). The lowest sound pressure level (SPL) that could generate an electrical response at the ABR thresholds of these different frequencies was considered. To evaluate if the lack of ACF7 has an impact on ABR thresholds, *Macf1^*fl/–*^; Pax2-Cre* mice were tested with control littermates under the same testing conditions. The intensities of SPL ranged from 100 dB SPL to 20 dB SPL, in 10 dB SPL intervals, and the responses were averaged over 1,024 sweeps. Tones were presented to mice via high-frequency transducers placed in the ear, with ABRs recorded in a soundproofed and closed, free-field system. ABR thresholds were determined post-procedure by identifying the lowest stimulus level that yielded a detectable ABR waveform at various time points.

### Statistics and Analysis

All statistics were performed with GraphPad Prism version 8. Data are reported as mean ± SEM or SD. Comparisons between groups were tested with unpaired two-tailed Student’s *t*-tests. R-Studio was used to generate circular histograms (RStudio, Inc.).

## Results

### Generation of a *Macf1* Conditional Knockout in the Ear

**FIGURE 1:**
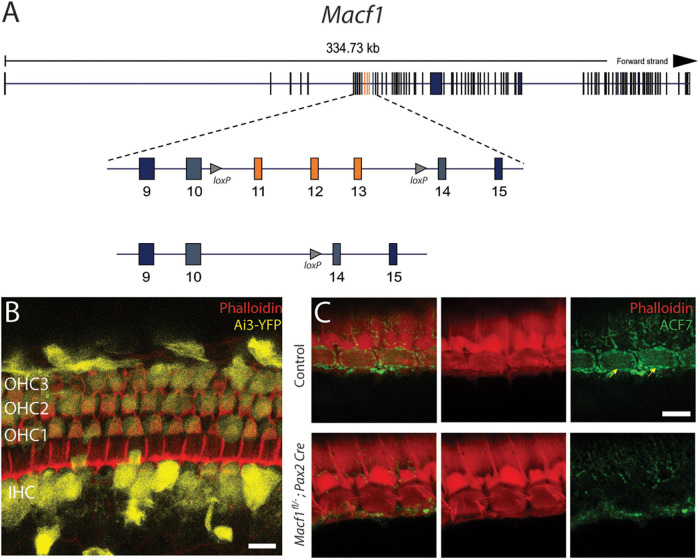
Conditional gene ablation of *Macf1* in mouse inner ear hair cells. **(A) (top)** Schematic of the *Macf1* genomic locus. **(middle)** Enlarged view of exons 9–15 are displayed. Exons 11–13 (orange) are flanked by *loxP* sites (triangles). **(B)** Representative confocal micrograph of a segment of the apical turn (∼8 kHz) of a cochlea from a Cre reporter Ai3-YFP mouse at one month of age that expresses Cre driven by the *Pax2* promoter, generated to determine the efficacy of Cre-mediated recombination in hair cells. Cells that express YFP (yellow) indicate successful Cre-mediated recombination. Hair cells of the cochlea express YFP. Phalloidin (red) labels actin of cells. Scale bar = 10 μm. **(C)** Representative confocal micrographs of IHCs from the middle turns (∼16 kHz) of cochleae of one-month old mice labeled with anti-ACF7 antibody [**(top)** sibling control; **(bottom)** *Macf1*^*fl*/–^; *Pax2*-*Cre*)]. Anti-ACF7 and phalloidin depicted in green and red, respectively. Arrows show cuticular plates with ACF7 antibody labeling. Hair bundles that appear missing are present but out of focus. Scale bar = 5 μm.

To understand the requirement of ACF7 in hair cells of mammals, we used a genetic approach. Because mice that lack ACF7 die pre-implantation (Kodama et al., 2003; Chen et al., 2006), we conditionally targeted the associated gene by using the genetically modified mouse *Macf1*^*fl/–*^, which has two *loxP* sites flanking a region encompassing exons 11 through 13 (Wu et al., 2008). This genetic strain has been successful in revealing the role of the ACF7 protein as a cytoskeletal integrator in other cell types as deletion of these exons are predicted to shift the coding sequence of downstream exons and has been shown to produce cells that are devoid of ACF7 (Fassett et al., 2013). In addition, we applied a strategy that used a *Pax2-Cre* allele as a Cre-driver line. Pax2 is expressed throughout the otic placode at E9.5 and thus should excise exons that are in-between *loxP* sites in all of the cells in the developing inner ear, including the hair cells in the cochlea (Ohyama and Groves, 2004; Figure 1).

Initially, to visualize Cre expression in the organ of Corti, *Pax2-Cre* mice were crossed with the Cre recombinase reporter Ai3-YFP (Madisen et al., 2010). In Ai3-YFP, transcription of the YFP reporter only occurs following Cre mediated excision of a premature stop codon. We observed YFP expression in most inner and outer hair cells (Figure 1B), indicating that the Pax2 driver has the spatial and temporal expression to knock out ACF7 in hair cells.

To determine if this stratagem was effective at eliminating ACF7 from hair cells, we labeled the organ of Corti of *Macf1^*fl/–*^; Pax2-Cre* and control mice with ACF7 antiserum and phalloidin to mark the actin of the cuticular plate (Karakesisoglou et al., 2000). In control inner hair cells, ACF7 localized to the cuticular plate and the circumferential band; however, the target protein was absent from inner hair cell cuticular plates and circumferential bands in the *Macf1^*fl/–*^; Pax2-Cre* mice, demonstrating that this gene targeting strategy was successful (Figure 1C). We did not observe labeling of outer hair cells in control and experimental groups. This could be because of different mounting procedures. Here, we use whole-mount labeling; however, previously we labeled cultured cochleae, indicating that labeling of outer hair cells may be sensitive to the immunological procedure (Antonellis et al., 2014). Since labeling of the cuticular plate and circumferential band is absent in inner hair cells, the conditional targeting strategy was effective.

### ACF7 Deficiency Does Not Affect Hair Cell Survival

**FIGURE 2:**
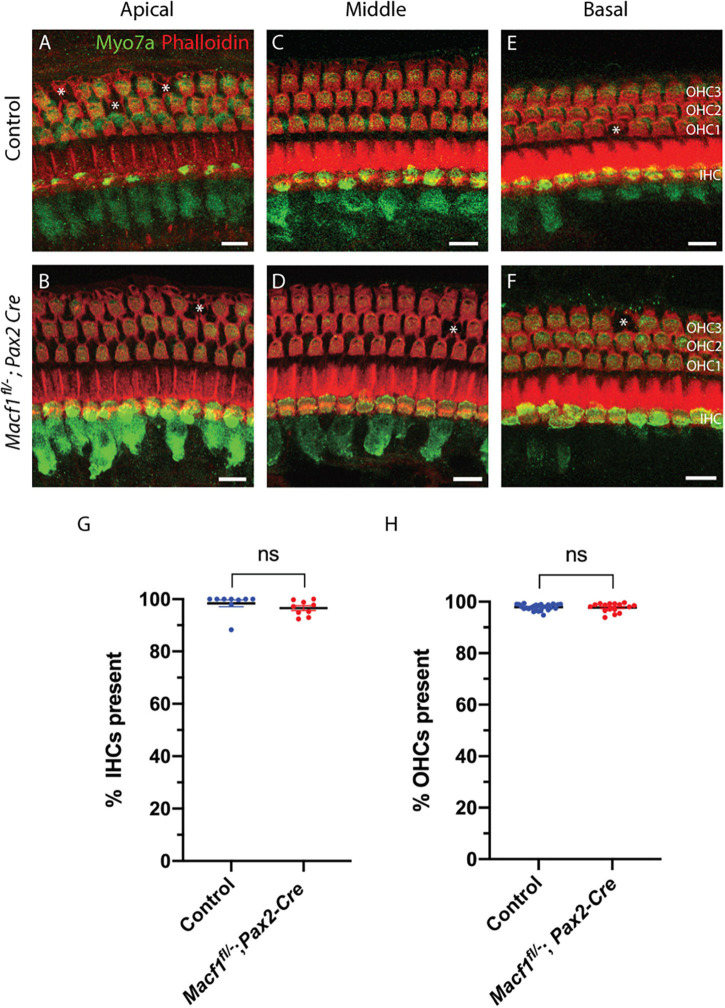
Hair cell viability is not impacted by conditional ablation of *Macf1*. **(A,C,E)** Micrographs of cochlear hair cells of control animals along apical **(A)** (*Macf1^*fl/+*^; Pax2-Cre*), middle **(B)** (*Macf1*^*fl/–*^), and basal **(C)** (*Macf1*^*fl/–*^) segments of the organ of Corti. **(B,D,F)** Images of cochleae from conditional knockout animals along the apical, middle, and basal segments. Myosin7a and phalloidin localization displayed in green and red, respectively. Each missing outer hair cell is indicated by an asterisk. **(G,H)** Mean percentages ± SEM of inner **(G)** (*p* = 0.26, *n* = 9 cochleae from 7 *Macf1*^*fl/–*^ mice, *n* = 9 cochleae from 6 *Macf1^*fl/*–^*; *Pax2-Cre* mice) and outer **(H)** (*p* = 0.76, *n* = 26 cochleae from 18 *Macf1*^*fl/–*^ mice, *n* = 16 cochleae from 11 *Macf1^*fl/*–^*; *Pax2-Cre* mice) hair cells present. Each data point represents one cochlea. Scale bar = 10 μm.

To determine if the presence of ACF7 is required for the survival of hair cells in any of the three turns of the cochlea, tissue of *Macf1^*fl/–*^; Pax2-Cre* and control mice were labeled for myosin7a and for actin using phalloidin (Figure 2). *Macf1^*fl/–*^; Pax2-Cre* mice are indistinguishable from their littermate controls, showing no apparent defects in the organ of Corti in either hair cells or supporting cells (Figures 2A–F). Quantification of hair cells between *Macf1^*fl/–*^; Pax2-Cre* and control littermates in one-month old mice revealed similar numbers of hair cells (Figures 2G,H), indicating that ACF7 is not required for hair cell survival in any of the cochlear turns.

### Hair Cell PCP Is Unperturbed in ACF7 Deficient Organ of Corti

**FIGURE 3:**
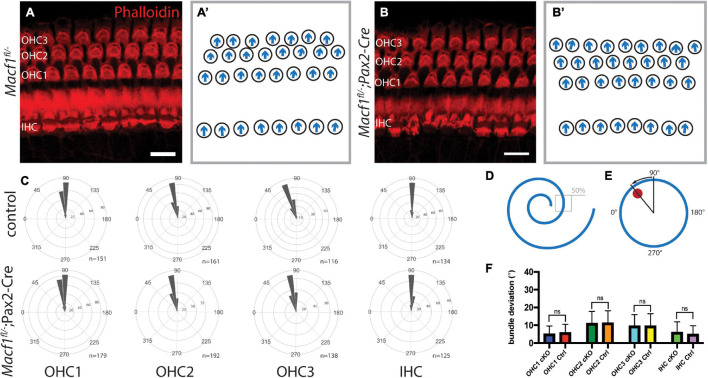
Auditory hair cell PCP is not affected in *Macf1^*fl/–*^; Pax2-Cre* mice. **(A,A′,B,B′)** Cochlear hair cells of one-month old mice, labeled with phalloidin (red), demonstrate hair bundle orientations. **(A,B)** Maximum projections of stacks of confocal images and **(A′,B′)** associated schematics of hair bundle orientations. The stacks are centered around the cuticular plate level and do not capture the full bundle lengths of all cells, but are meant to reveal PCP only. **(C)** Orientations of stereociliary bundles plotted on circular histograms for all hair cells from the middle turn of the cochlea (∼50% cochlear length). 0° is directed toward the apical turn of the cochlea, and 180° is toward the base. Bin width is 10°. The total number of represented hair cells is shown by each histogram (*n*). Scale bars = 10 μm. **(D)** Schematic indicating the region located at ∼50% along the length of a cochlea for stereociliary bundle orientation analyses. **(E)** A representative schematic of a single hair bundle demonstrating the angular measurement for quantifying stereociliary bundle deviation. The red dot marks the position of the kinocilium, and the arrow shows the deviation. **(F)** Averaged bundle deviation from the mediolateral axis of a hair bundle for *Macf1*^*fl/–*^; *Pax2-Cre* (cKO) and control mice. For orientation and bundle deviation assays, the number of mice analyzed is *n* = 5 for *Macf1*^*fl/–*^; *Pax2-Cre* and *n* = 4 (*n* = 2 for *Macf1*^*fl/–*^, *n* = 2 for *Macf1^*fl/+*^; Pax2-Cre*) for controls. Error bars show standard deviation. There is no statistical significance between *Macf1*^*fl/–*^; *Pax2-Cre* and control mice. Calculated using a two-tailed Student’s *t*-test (*p* = 0.129 for OHC1, *p* = 0.678 for OHC2, *p* = 0.959 for OHC3, *p* = 0.110 for IHC).

Since ACF7 has a compelling localization pattern in and around the cuticular plate, we tested the hypothesis that this protein may be involved in PCP. *Macf1*^*fl/–*^; *Pax2-Cre* mice and littermate controls were examined for defects in PCP by labeling actin followed by a hair bundle orientational assay on the organ of Corti (Figures 3A,A′,B,B′). The orientation of individual stereociliary bundles at the approximate midpoints along the lengths of the cochleae (Figures 3D,E) were measured and plotted in circular histograms (Figure 3C). Average bundle deviation from the midlateral axis of hair bundles across different animals were measured for *Macf1*^*fl/–*^; *Pax2-Cre* mice and control mice (Figure 3F). In each row, OHC1, OHC2, OHC3, or IHC, there was no significant difference in the average stereociliary bundle deviation between the *Macf1*^*fl/–*^; *Pax2-Cre* mice and the controls.

### ACF7 Deficiency Reduces Kinocilium Length in Postnatal Mice

**FIGURE 4:**
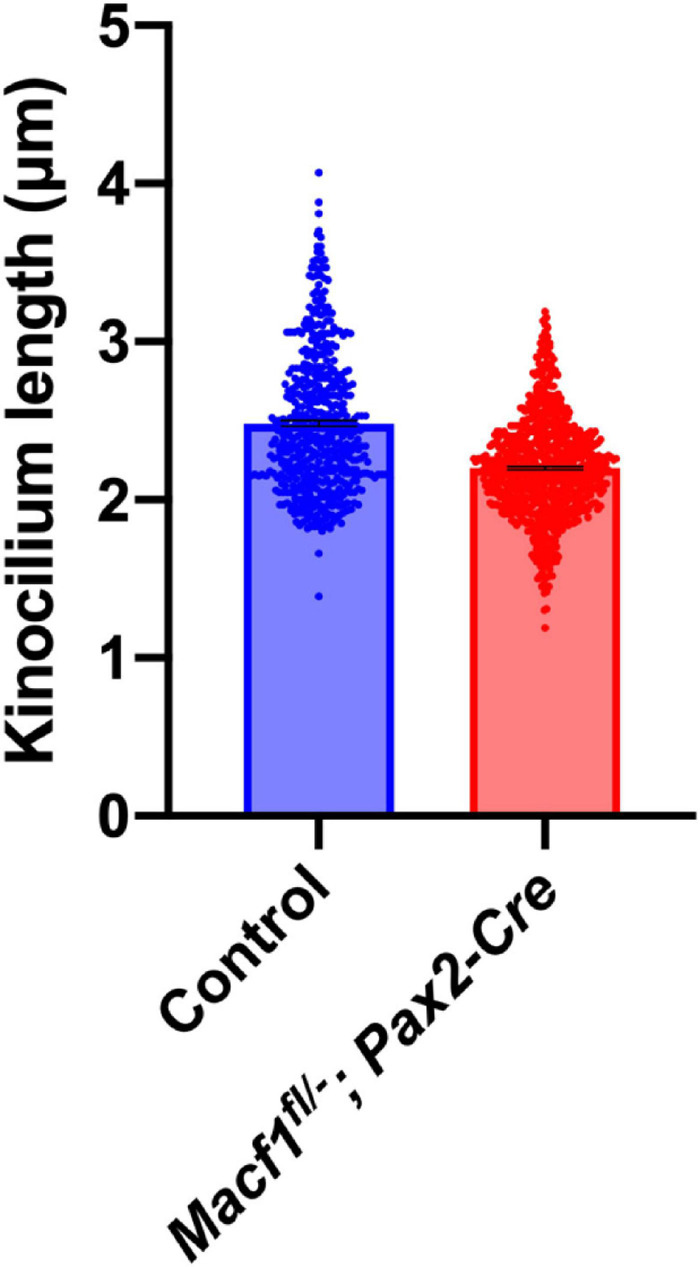
Kinocilium length measurements of *Macf1^*fl/–*^; Pax2-Cre* and sibling control mice. Bar graph depicts mean lengths of outer hair cell kinocilia from the apical cochlear turns of P5 mice. The control group’s (blue; *Macf1*^*fl/–*^) mean kinocilium length is 2.49 ± 0.02 μm (*n* = 527 hair cells from 3 cochleae from 3 mice). The mean length for the conditional knockout group (red) is 2.20 ± 0.01 μm (*n* = 766 hair cells from 3 cochleae from 3 mice). *P* < 0.0001 calculated using an unpaired Student’s *t*-test.

In prenatal mice, ACF7 is required for normal kinocilia lengths (May-Simera and Kelley, 2012). Therefore, we tested if kinocilia lengths were altered in P5 *Macf1*^*fl/–*^; *Pax2-Cre* mice, a timepoint during cochlear development that was chosen because it is before the kinocilia degenerate (Wang and Zhou, 2021) but should still also reflect defects in kinocilia formation if they do exist in these conditional knockout animals. Using anti-acetylated tubulin to label the kinocilia and confocal microscope software for measurements, we showed that the mean length of kinocilia from control mice is 2.49 ± 0.02 μm (mean ± SEM) (Figure 4). In contrast, the mean length of kinocilia from the *Macf1*^*fl/–*^; *Pax2-Cre* mice is 2.20 ± 0.01 μm. The significant reduction of mean kinocilium length in the conditional knockout mice demonstrates that ACF7 is required for normal kinocilium length after birth.

### ACF7 Deficiency Does Not Affect Hearing

**FIGURE 5:**
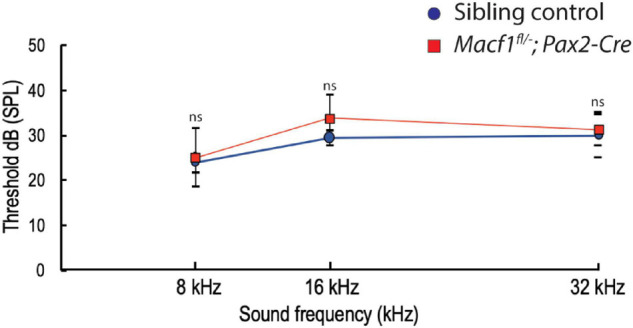
Auditory brainstem response (ABR) thresholds of *Macf1^*fl/–*^; Pax2-Cre* and sibling control mice. Thresholds at 8, 16, and 32 kHz show no statistical significance between *Macf1^*fl/–*^; Pax2-Cre* mice and controls. *n* = 5 control mice and *n* = 6 *Macf1^*fl/–*^; Pax2-Cre* mice. Error bars are represented as the standard deviation and significance was determined with an unpaired Student’s *t*-test. dB, decibels.

To further investigate whether the lack of ACF7 has an impact on inner hair cell function, we compared ABR thresholds of *Macf1^*fl/–*^; Pax2-Cre* mice to littermate control mice. We observed no significant difference (Figure 5) in ABR thresholds between the two groups at one-month of age. ABR thresholds (Mean ± SEM) for the control group were 23.9 ± 2.2 (8 kHz), 29.4 ± 1.7 (16 kHz), and 30.0 ± 5 (32 kHz); in contrast, ABR thresholds for the *Macf1*^*fl/–*^; *Pax2-Cre* group were 25.0 ± 6.6 (8 kHz), 33.8 ± 5.2 (16 kHz), and 31.3 ± 3.5 (32 kHz).

## Discussion

In the present study, the role of ACF7 in hair cell structure and function was examined using the inner ear-specific conditional knockout mouse *Macf1*^*fl/–*^; *Pax2-Cre*. Our previous data showed that in the hair cells of zebrafish and mice, ACF7 displays a compelling localization pattern in and around the cuticular plate (Antonellis et al., 2014). The expression pattern of ACF7 in hair cells combined with its role as a cytoskeleton integrator leads to the hypothesis that ACF7 is an essential hair cell protein. To our surprise, our studies showed that ACF7 deficiency does not appear to negatively impact hair cell integrity or function beyond a modest reduction in the kinocilium length, similar to what was initially reported by May-Simera et al., 2016. In agreement with these findings, young, but mature, *Macf1*^*fl/–*^; *Pax2-Cre* mice do not exhibit hearing defects.

A recent study investigating the role of ACF7 in platelets using *Macf1*^*fl*^ to conditionally knockout the gene also revealed that ACF7 was not required for proper function in certain contexts. These findings are relevant because, similar to hair cells, platelets have a robust cytoskeletal structure that is required for the cell to survive and function properly (Schurr et al., 2019). This suggests that both hair cells and platelets have possible redundancy of the cytoskeleton proteins to safeguard their precise structure that is essential for their function. A suitable candidate with potential redundant function in hair cells is the other member of the spectraplakin family, bullous pemphigoid antigen 1 (BPAG1), which shares a similar primary amino acid sequence similarity with ACF7 and is also expressed in cochlear hair cells (Leonova and Lomax, 2002; Voelzmann et al., 2017). *Bpag1* mutations can yield phenotypes similar to those of *Macf1* mutants (Suozzi et al., 2012; Ali et al., 2017). On a cellular level, *Bpag1* mutation is primarily categorized by severe cytoskeletal disruption in a plethora of tissues, producing distinctive neurological, muscular, and skin conditions. Interestingly, a human patient was recently identified with compound mutations for *BPAG1* that additionally resulted in the development of early and progressive bilateral hearing loss, suggesting that BPAG1 may in fact be relevant to hearing (Cappuccio et al., 2017). Given our data and the literature, it is plausible that the expression of BPAG1 in cochlear hair cells rescues the cells from what would otherwise perturb hair cells in the conditional *Macf1* knockout.

There are other possible explanations as to why there is no observable hair cell phenotype in *Macf1*^*fl/–*^; *Pax2-Cre* mice beyond a modest reduction in kinocilium length. First, it is possible that ACF7 is not required for hair cell structure and function at all. It is also plausible that ACF7 plays a role during a small window of time during development, and there are deficits that exist during this period; however, there is recovery sometime afterward, similar to what has been observed in mutant mice that have defects in factors that govern PCP (Copley et al., 2013). Additionally, it may be that ACF7 plays a more protective role in mature hair cells, rather than a function during development. Acoustic over stimulation has the potential to permanently damage the hair cell stereocilia and hair cell function. Perhaps the localization of ACF7 to the cuticular plate provides stability to the rootlets of the stereocilia in order to render some degree of resilience to the stereocilia bundles against noise-induced damage. Additionally, the loss of ACF7 in conditional mutants may not have physiological impacts until later in life and cause the early onset of age-related hearing loss. These hypotheses could be tested in the future by evaluating the resistance of conditional knockout *Macf1* mice to noise-induced hearing loss and by studying mice at more mature time points.

In summary, there are several possible functions for ACF7 in the hair cells of mice; however, evidence to support a role for ACF7 in cochlear hair cell development and function beyond the kinocilium is lacking for the time points examined. Nevertheless, our study showed that a compelling localization pattern of a protein in hair cells may not equate to a critical role for that protein in the cell; examples in the literature are consistent with this. Specifically, annexin A5 is one of the most abundant proteins in stereocilia; however, it was found to be dispensable for stereocilia development and function (Krey et al., 2016). The purpose of ACF7 expression in hair cells beyond a role in kinocilium length remains to be understood.

## Data Availability Statement

The raw data supporting the conclusions of this article will be made available by the authors, without undue reservation.

## Ethics Statement

The animal study was reviewed and approved by the Case Western Reserve University Institutional Animal Care and Use Committee.

## Author Contributions

BG and SZ: fluorescence labeling and imaging. BG, AS, and SS: genetics. AS, BG, SZ, KA, and BM: experimental design. AS and BG: ABRs. BG, SZ, AS, KA, and BM: manuscript writing and editing. All authors contributed to the article and approved the submitted version.

## Conflict of Interest

The authors declare that the research was conducted in the absence of any commercial or financial relationships that could be construed as a potential conflict of interest.

## Publisher’s Note

All claims expressed in this article are solely those of the authors and do not necessarily represent those of their affiliated organizations, or those of the publisher, the editors and the reviewers. Any product that may be evaluated in this article, or claim that may be made by its manufacturer, is not guaranteed or endorsed by the publisher.

## Supplementary Material

Supplementary Figure 1 Representative whole-mount immunofluorescence on cochleae from experimental and control mice. **(A,B)** *Macf*1^*fl*/−^; *Pax2-Cre* and control cochleae labeled with phalloidin (red) and a myosin7a antibody (green). For display, each whole cochlea divided in two is shown minus the hook region. **(C)** Schematics of cochlear regional boundaries. Incisions were made to sever the apical and basal regions from the middle turn. The cochleae were divided isometrically. Scale bar 50 μm.
