# Supplementary figures and images for: Actin Crosslinking Family Protein 7 Deficiency Does Not Impair Hearing in Young Mice
Source: Front Cell Dev Biol. 2021 Nov 30;9:709442. doi: 10.3389/fcell.2021.709442 (PMC8670236; doi:10.3389/fcell.2021.709442)

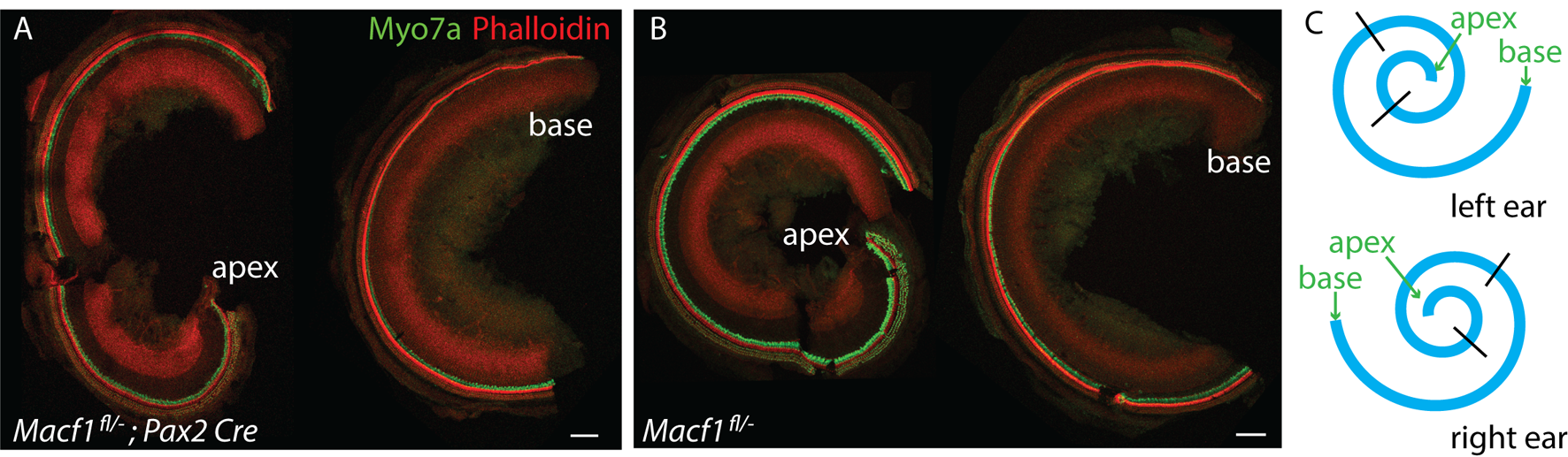

Supplement: Supplementary Figure 1 — Representative whole-mount immunofluorescence on cochleae from experimental and control mice. (A,B) Macf1fl/−; Pax2-Cre and control cochleae labeled with phalloidin (red) and a myosin7a antibody (green). For display, each whole cochlea divided in two is shown minus the hook region. (C) Schematics of cochlear regional boundaries. Incisions were made to sever the apical and basal regions from the middle turn. The cochleae were divided isometrically. Scale bar 50 μm. [file Image_1.TIF]
